# Supplementary material for: A Residual N-Terminal Peptide Enhances Signaling of Depalmitoylated Hedgehog to the Patched Receptor
Source: J Dev Biol. 2024 Apr 9;12(2):11. doi: 10.3390/jdb12020011 (PMC11036296; doi:10.3390/jdb12020011)
Supplement: Supplementary file 1 [file jdb-12-00011-s001.zip › jdb-2937158-supplementary.pdf]

Table S1: Information regarding amplicons and primers

abbreviation	isoform	FWD primer (5'-3')	REV primer (3'-5')	name
**Actin β**	1	CTATTGGCAACGAGCGGTTC	CGGATGTCAACGTCACACTTC	Actin
**Ptch1**	2	GGGCTACGACTATGTCTCTC	CTTTGATGAACCACCTCCAC	Protein patched homolog 1
**Gli1**	1	CCCTGGTGGCTTTCATCAAC	TGACTCATCTGAGGTGGGAATC	Zinc finger protein GLI1
**Gli2**	1	CAACTCAGCAGCAGTAGCAG	CTCCGCTTATGAATGGTGATGG	Zinc finger protein GLI2
**Gli3**	1	GTGGTTCCTATGGGCACTTATC	GTCGGCTTAGGATCTGTTGATG	Zinc finger protein GLI3
**Dlk1**	4	GGCTGTGTCAATGGAGTCTG	AAGCCCGAACGTCTATTTCG	Delta like non-canonical Notch ligand 1 (= Pref1)
**Pparg**	4	TCCATTCACAAGAGCTGACC	GGTGGAGATGCAGGTTCTAC	Peroxisome proliferator-activated receptor γ
**Fabp4**	1	GTGTGATGCCTTTGTGGGAAC	CATGCCTGCCACTTTCCTTG	Fatty acid-binding protein (= Ap2)
**Cfd**	3	CCTGAACCCTACAAGCGATG	CAACGAGGCATTCTGGGATAG	Complement factor D (= adipsin)
**Dgat2**	1	GGCTGATAGCTGTGCTCTAC	GATGGGAAAGTAGTCTCGGAAG	Diacylglycerin-acyltransferase 2
**Alpl**	5	CTGCAAGGACATCGCATATCAG	CCACATCAGTTCTGTTCTTCGG	Alkaline phosphatase (pan-expressed)
**Spp1**	5	ACAGAATGCTGTGTCCTCTG	GGTCTCCATCGTCATCATCATC	Secreted phosphoprotein 1 (= Osteopontin)
**Bglap**	2	CCAAGCAGGAGGGCAATAAG	CTCGTCACAAGCAGGGTTAAG	Bone γ-carboxy-glutamate protein (Osteocalcin)
**Runx2**	6	ACACTGCCACCTCTGACTTC	GGGATGAAATGCTTGGGAACTG	Runt related transcription factor 2
**Sox9**	1	CGGAACAGACTCACATCTCTCC	GACCCTGAGATTGCCCAGAG	SRY (sex determining region Y)-bos 9
**Col2a1**	2	CTGAAGGTGCTCAAGGTTCTC	GATCCTTTGGCTCCAGGAATAC	Collagen type II α I
**Col10a1**	1	TCTCCCAGCACCAGAATCTATC	CCATGAACCAGGGTCAAGAAC	Collagen type X α I
**Col1a1**	1	TGGTCCACAAGGTTTCCAAG	CATCTCCATTCTTGCCAGGAG	Collagen type I α I
**Mmp3**	1	ACTTGTCCCGTTTCCATCTC	GGTTCCAGAGAGTTAGACTTGG	Matrix metallopeptidase 3
**Cdk9**	1	CAGCTCTGTGGCTCCATCAC	GTCCTTCACCTTCCGCTTCTG	Cyclin-dependent kinase 9 (CDC2-related kinase)
**Mki67**	1	TGAGGCTGAGACATGGAGAC	GGTTCCTTTCCAAGGGACTTTC	Antigen identified by monoclonal antibody Ki67
